# Prevalence and correlates of *Schistosoma haematobium* infections among school going-children aged 5 to 17 years in Kawama, Ndola, Zambia

**DOI:** 10.11604/pamj.2023.45.170.41193

**Published:** 2023-08-18

**Authors:** Chileshe Sandema, Victor Daka, Paul Syapiila, Mathias Tembo, Jay Sikalima, Shivangi Patel, Steward Mudenda, Ruth Lindizyani Mfune, Imukusi Mutanekelwa, Cosmas Zyambo, Victor Mwanakasale

**Affiliations:** 1 Copperbelt University, Michael Chilufya School of Medicine, Public Health Department, P.O Box 71191, Ndola, Zambia,; 2 Tropical Diseases Research Centre, Biomedical Sciences Department, P.O Box 71769, Ndola, Zambia,; 3 Churches Health Association of Zambia, Molecular Laboratory Department, P.O.BOX 34511, Lusaka, Zambia,; 4 University of Zambia, School of Health Sciences, Department of Pharmacy, P.O Box 50110, Lusaka, Zambia,; 5 University of Zambia, School of Public Health, Department of Community and Family Medicine, P.O. Box 50110, Lusaka, Zambia,; 6 Copperbelt University, Michael Chilufya Sata School of Medicine, Basic Sciences Department, P.O Box 71191, Ndola, Zambia

**Keywords:** *Schistosoma haematobium*, Schistosomiasis, school children, Kawama, Ndola

## Abstract

**Introduction:**

schistosomiasis is a neglected tropical disease and remains a disease of public health concern. Despite its relative importance, paucity of information on schistosomiasis in urban settings such as Ndola remains. Here, we present findings on the prevalence and factors associated with Schistosoma haematobium (S. haematobium) infections among School-going children in the Kawama in Ndola district in Zambia, an urban area in the Copperbelt Province, Zambia.

**Methods:**

we employed a cross-sectional study design among 354 school going-children between 5 and 17 years of age between November 2020 and February 2021. A Multivariate forward step-wise logistic regression model was used to determine the associations of risk factors. Adjusted odds ratios and 95% confidence intervals are reported.

**Results:**

of the 354 school-going children included in the analysis, 13.3% had S. haematobium infection. Children who swam in the stream/dam were more likely to have S. haematobium infection as compared to those who did not (aOR 6.531, 95% CI: 2.90-14.69).

**Conclusion:**

S. haematobium infection is endemic among school-going children in an urban setup of the Kawama area of Ndola City, Zambia. There is a need for targeted interventions to mitigate infections among this population.

## Introduction

Schistosomiasis which is also known as bilharzia is a disease caused by species of blood trematodes (flukes) that belong to the genus *Schistosoma* [1]. Schistosomiasis is the second most common neglected tropical disease in sub-Saharan Africa [2,3].

Individuals can get infected during agricultural, occupational, domestic, and recreational activities, which involve contact with water bodies infested with schistosome-infected snails. These water bodies include slow-moving waters of tropical rivers, lakes, streams, and irrigation ditches [4]. School-going children aged 5 to 15 years have been known to have an increased risk of *Schistosoma haematobium (S. haematobium)* infection [5-7]. Other groups that are at risk include adults in endemic areas, entire communities living in highly endemic areas, and people with occupations involving contact with water bodies infested with parasites like fishermen, farmers, irrigation workers, as well as women doing routine domestic tasks [8]. Unhygienic and risky playing habits among children such as swimming and/or fishing in infested water make them vulnerable to infection [9-11]. Furthermore, water contact activities, location, and mothers' occupations have been found to be significantly associated with urogenital schistosomiasis [12,13]. Water bodies are a source of snail hosts with fishing and working in gardens along the river being potential risk factors for *S. haematobium* infection [14]. Infection with schistosomiasis has economic and health impacts at the household level. Chronic schistosomiasis may be debilitating affecting people´s ability to work [15]. Another health effect of schistosomiasis includes genital lesions which heighten the risk of contracting other diseases such as HIV and cancer of the cervix or bladder [16].

In Zambia, the risk of *Schistosoma* infection has increased and has been attributed to environmental, socioeconomic, and demographic factors. Environmental changes may have a predilection for the alteration of infection risk driven by changes in the ecology of snail hosts [17]. There have been increased reports of schistosomiasis from the Kawama clinic in Ndola, Zambia [18]. In addition, we postulate that the presence of water bodies in this area could be a critical driver of these cases. To understand the magnitude of this problem and its drivers, we conducted a study to investigate the prevalence of schistosomiasis among school-going children in this area and possible risk factors.

## Methods

**Study site:** the study was conducted in Kawama of the Ndola district of the Copperbelt Province in Zambia from November 2020 to February 2021. Kawama has a population density of 14.250 per km^2^and 3,402 households. Children account for 75% of the total population [19]. The compound is a waterlogged area with a lot of stagnant water which residents use for agricultural and recreational activities. The study included pupils from all four schools in Kawama township (Kawama primary school, Garden of Hope primary school, Intulo primary school and Joy community school in Kawama). All laboratory analyses were done at the Kawama clinic.

**Study population and study design:** this was a cross-sectional study. Pupils from grades 2 to 7 at the selected schools had an equal chance of participating in the study.

**Sample size determination:** we assumed a prevalence (p) of 22.6% obtained from a previous study conducted in the Chifubu area of Ndola [20]. Chifubu area is approximately 3 kilometres from Kawama with similar population and geographical characteristics and hence would not be affected by clustering. The sample size was estimated using Cochrane's formula [21];


$$n=\frac{Z^{2}px\left(1-p\right)}{d^{2}}$$

At 95% confidence level, where n is the required sample size, d is the margin of error estimated at 5% and z is the z-score from the normal distribution given as 1.96. Employing a 20% non-response we estimated the minimum sample size of 323.

**Sampling procedure:** a systematic random sample was used to select children from grades 2 to 7 to participate in the study from the four selected schools. The number of pupils selected from each school was proportional to the cumulative number at each school.

**Data collection and tools:** only pupils with consent forms signed by their parents or guardians and willing to participate in the study were enrolled. Pupils enrolled on the study were given a urine specimen bottle and asked to void urine in it. A closed-ended questionnaire was administered to the pupil by the research assistant to collect demographic information (part A) and water contact activities (part B). The Global Positioning System (GPS) coordinates of homes of participants who tested positive with *S. haematobium* were taken to show the spatial distribution of schistosomiasis in Kawama Township, Ndola. All collected urine specimens were transported to the Kawama Clinic laboratory for analysis. Ten (10) ml of urine samples were collected from participants. The urine concentration method was used. Briefly, the specimen was centrifuged at 1500 rpm for 2 minutes, the supernatant was decanted, the deposit was re-suspended and a drop of the deposit was placed onto a well labelled clean grease-free microscopic slide and covered with a cover slip. The deposit was examined using the X10 and then X40 objectives lenses to determine the presence of *S. haematobium* infection.

**Data analysis:** questionnaires were checked for completeness and consistency in Excel. The data were exported to SPSS version 20 (IBM Corp., Armonk, NY, USA) for statistical analysis. Frequency tables were used for descriptive statistics and a spot map indicating residential areas of participants was used to show the spatial distribution of schistosomiasis. We used a Stepwise Forward Logistic regression and reported odds ratio to measure the association and strength between variables. Univariate analysis was done via Chi-square and all statistically significant variables at p value 0.05 were included in the multivariate analysis. Adjusted odds ratios and their corresponding 95% confidence intervals were reported. Finally, for all analyses, a p-value of 0.05 denoted statistical significance. Missing data was handled during data analysis via listwise deletion.

**Reliability and validity of the questionnaire:** a pilot study was used to test the reliability and validity of the data collection tool. The study population was the school-going children in Chifubu, Ndola, a similar area to Kawama. The pilot study was done among 30 participants and the data collected in the pilot did not form part of the main study. Results and observations from the pilot study were used to optimise the validity and reliability of the questionnaire for the main study.

**Ethical consideration:** ethical clearance for this study was approved by the Tropical Disease Research Centre Ethics Committee (IRB Registration number: 00002911). Permission was also obtained from the District Health Office, Ministry of Education- Ndola District Education Board Secretary (DEBS) and National Health Research Authority (NHRA) (Ref No: NHRA0002/2/03/2021). Consent forms were given to pupils through the class teachers together with a detailed description of the study objectives, potential risks and benefits. Only pupils whose parents signed the consent forms were enrolled in this study. All pupils were found to be positive for *S. haematobium* infection was referred to Kawama clinic for management. These pupils were given a single dose of praziquantel at 40 mg/kg body weight. The study was of minimal risk to participants and all data collected in the study were restricted to the investigators. No identifiers were used on specimens and questionnaires and linkage to individual participants was only done for linkage to routine care for further management where needed.

## Results

A total of 354 pupils were enrolled into the study from a total number of 2150 eligible pupils. There were more male participants (53.1%) than females (46.9%). Approximately 189 (53.4%) were aged between 5 and 10 years while only 3(0.8%) were above the age of 15 years. Most participants were from grade two 151 (42.7%) while the least was in grade 7 (3.7%) (Table 1). Of the 354 pupils who participated in the study, 47 tested positive for schistosomiasis giving an overall prevalence of 13.3%. The highest prevalence was recorded in males (19.1%) and the age group 11-15 years (19.8%).

**Table 1:** demographic characteristics of participants

Demographic factors	Number of participants	Percentage (%)
**Sex**		
Male	188	53.1
Female	166	46.9
**Age of participants (years)**		
5-10	189	53.4
11-15	162	45.8
> 16	3	0.8
**Grade**		
2	151	42.7
3	31	8.8
4	50	14.1
5	50	14.1
6	59	16.7
7	13	3.7
**Primary school**		
Chawama primary school	77	21.8
Intulo primary school	183	51.7
Joy community school	36	10.2
Garden of hope community school	58	16.4
**Total**	**354**	**100**

After screening independent variables for possible association with schistosomiasis (dependent variable) using a Chi-square, sex (p = 0.001), age group (p = 0.004), grade (p = 0.001), playing in the steam/dam (p < 0.001), swimming in the stream/dam (p < 0.001) and fishing in the stream/ dam (p < 0.001) showed a statistically significant association. These factors were therefore included in multivariate logistic regression analysis to come up with a final predictive model for schistosomiasis in Kawama Township, Ndola (Table 1).

**Figure 1:**
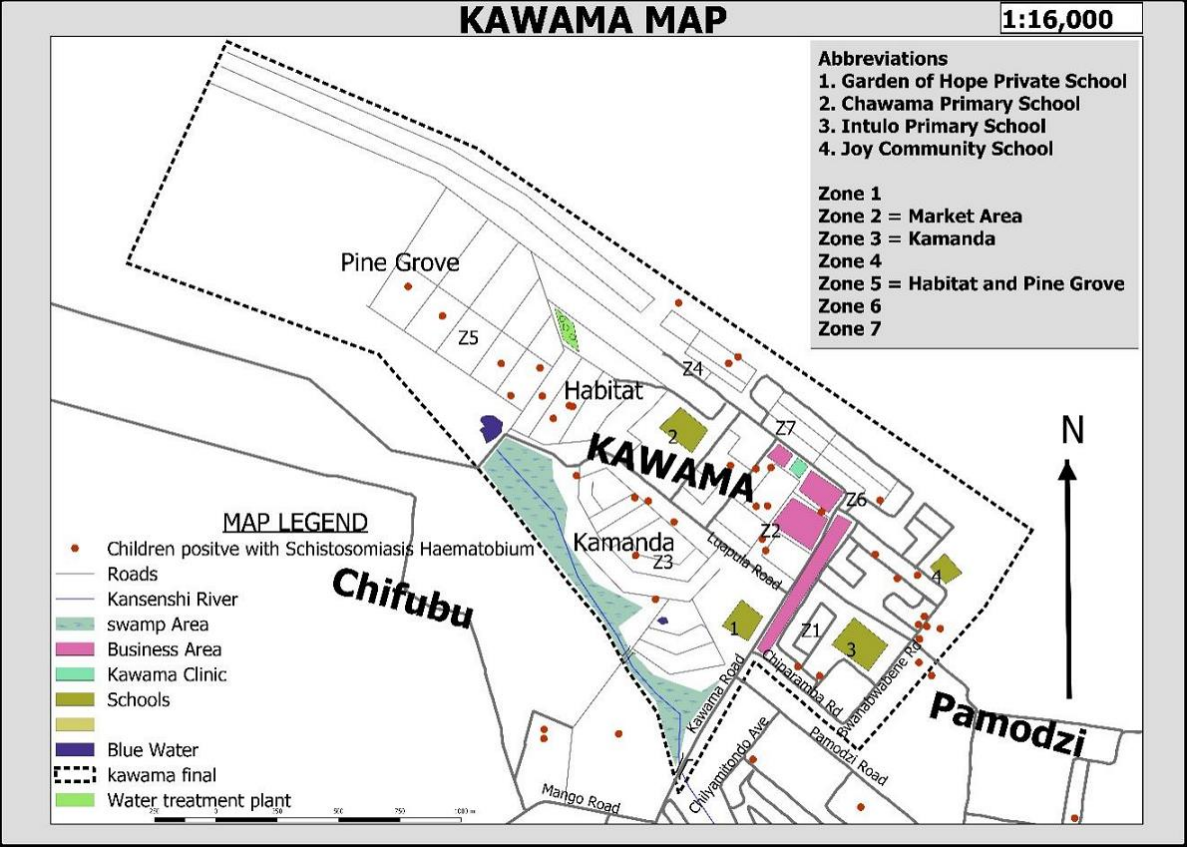
spatial distribution of schistosomiasis cases in Kawama

After adjusting for possible confounding, swimming in the stream/dam (Adjusted Odds Ratio (AOR) = 6.531, 95% Confidence Interval (CI): 2.90-14.69) was the water-related activity that was independently associated with schistosomiasis in Kawama. Fishing in the stream/dam (p < 0.001) showed a statistically significant association in bivariate analysis but was not significant in multivariate analysis (p = 0.053). Participant´s school grade was also found to be associated with schistosomiasis with grades four (AOR 3.580, 95% CI 1.27- 10.12), grade six (AOR 7.349, 95% CI 2.70-20.00) and grade seven (AOR 9.008, 95% CI 1.91-42.49) showing a statistically higher likelihood of having schistosomiasis than grade twos. Being in grade three and grade five. However, did not statistically show any excess risk compared to being in grade two (Table 1). From the spatial distribution of schistosomiasis cases in Kawama Township as shown in Figure 1, cases seem to have been evenly distributed throughout the township.

**Table 2:** bivariate analysis of factors associated with schistosomiasis

Risk factors	Cases (%)	None cases (%)	Chi-square	P-value
**Sex**				
Male	36 (19.1)	152 (80.9)	1	
Female	11 (6.6)	155 (93.4)	12.006	0.001
**Age group (years)**				
5-10	15 (7.9)	174 (92.1)		
11-15	32 (19.8)	130 (80.2)	11.042	0.004
> 16	0 (0.0)	3 (100)		
**Grade**				
2	9 (6)	142 (94.0)		
3	3 (9.7)	28 (90.3)		
4	10 (20)	40 (80)	20.418	0.001
5	6 (12)	44 (88)		
6	15 (25.4)	44 (74.6)		
7	4 (30.8)	9 (69.2)		
**Swimming**				
Yes	35 (29.2)	85 (70.8)		
No	12 (5.1)	222 (94.9)	39.809	<0.001
**Gardening near stream/dam**				
Yes	3 (5.5)	52 (94.5)		
No	44 (14.7)	255 (85.3)	3.460	0.063
**Washing in the stream/dam**				
Yes	13 (16.7)	65 (83.3)		
No	34 (12.3)	242 (87.7)	0.998	0.318
**Fishing in the stream/dam**				
Yes	17 (37.0)	29 (63.0)		
No	30 (9.7)	278 (90.3)	25.747	<0.001
**Drawing water from stream/dam**				
Yes	11 (17.2)	53 (82.8)		
No	36 (12.4)	254 (87.6)	1.038	0.308
**Washing plates from the stream/dam**				
Yes	12 (21.1)	45 (78.9)		
No	35 (11.8)	262 (88.2)	3.568	0.059
**Total**	**47 (13.3)**	**307 (86.7)**		

**Table 3:** multivariate analysis of factors associated with schistosomiasis

Factors	Adjusted odds ratio (AOR)	95% confidence interval	
		Lower limit	Upper limit
**Demographic factors**			
**Sex**			
female	1		
male	1.632	0.71	3.74
Participant’s grade			
Grade 2	1		
Grade 3	2.515	0.58	10.90
Grade 4	3.580	1.27	10.12
Grade 5	2.671	0.82	8.73
Grade 6	7.349	2.70	20.00
Grade 7	9.008	1.91	42.49
**Water-related activities**			
Residence near a water body	1		
Swimming	6.531	2.90	14.69
Fishing	2.261	0.99	5.17

## Discussion

The current study investigated the prevalence of schistosomiasis among school-going pupils from Kawama township of Ndola district in Zambia. This study shows that the prevalence of *S. haematobium* among school-going children in Kawama Township was 13.3%. This is similar to findings from another study that reported 14% in North Western Province but higher (61%) in another study in Luapula Province [22,23]. Higher prevalence rates have been reported in other countries including 20.8% in North Eastern Ethiopia [24] and 45.6% in Kwara state in Nigeria [25]. Similarly, higher prevalence rates have been reported from rural Norther Ghana (33.2%), Magba subdivision of Cameroon (41.1%) and Yemen (23.8%) [26-28]. A lower prevalence of 3.7% was reported in a study done in the Altakamol area, Khartoum state, Sudan among primary school-going children [29] while less than one percent positivity for *S. haematobium* was reported in a study done in villages surrounding Lake Nyasa [30]. A study done in Um-Asher Area, Khartoum, Sudan reported a prevalence of 12.9% [31], findings that are in agreement to those in the current study among school-going children in Kawama, Ndola. Rural areas have a higher prevalence compared to urban areas in Zambia. These differences in the prevalence of *S. haematobium* reported in different communities could be attributed to differences in the distribution of predisposing factors such as swimming in water bodies contaminated with *Schistosoma* infected snails and the coverage and effectiveness of public health interventions against the parasite. Additionally, differences in socio-economic status between rural and urban areas could be a driver of infection [32].

This study found that a pupil´s grade was associated with *S. haematobium* infection in Kawama Township. Participants in grades four, six, and seven showed a statistically higher likelihood of having *S. haematobium* infection than those in grade two. Being in grade three and grade five, however, did not statistically show any excess risk compared to being in grade two. Sex was not found to be statistically significant in multivariate analysis. However, in bivariate analysis, male participants were more likely to be infected (19.1%) with *S. haematobium* than their female counterparts. The findings are consistent with results reported in other studies [25,27,29,33,34]. In contrast, two previous studies found no significant difference in *Schistosoma* infection between males and females [24,31]. This could be explained by the fact that different communities have different activities for male and female children which could predispose them to *S. haematobium* infection differently.

Our study established that water contact activities like swimming in the stream/dam were significantly associated with *S. haematobium* infection. Similar findings have been reported in Ivory Coast [35], Ghana [26], Nigeria [25,33], and Zambia [22]. On the contrary, other water contact activities like fishing in the stream/dam, gardening along the stream/dam, washing plates or clothes in the stream/dam, and playing and drawing water in the stream/dam were not found to be significantly associated with *S. haematobium* infection in this study.

Being a resident near water bodies has been reported to increase the risk of *S. haematobium* infection [5,26,36,37]. However, in this present study, the cases were evenly distributed across Kawama township with no visible clustering around water bodies. This could mean that all the children in Kawama had equal access to the water bodies despite the differences in the proximity of their residential areas to the water bodies. Hence, the need to educate pupils on the dangers of playing in various water bodies.

**Study limitation:** our study was limited to the Kawama area in Ndola and findings from this study may not be representative of other populations. In addition, our study may be limited in the scope of risk factors investigated as only three were added to the regression model. However, this is the first study to investigate schistosomiasis in this area and our findings are key in formulating control measures.

## Conclusion

Schistosomiasis is a public health problem in Kawama Township with a prevalence of 13.3% among pupils aged 5 to 17 years. Water contact activities like swimming propagate its transmission. Pupils in grades four, six, and seven are more likely to be infected with *S. haematobium*.

**Recommendations:** there is a need for interventions such as targeted preventive chemotherapy and health education on the risk factors associated with schistosomiasis. We recommend annual treatment of all school-going children with a single dose of praziquantel at 40 mg/kg body weight. In addition to this, swimming in cercariae-infested water should be heavily discouraged and/or prohibited. Health education should be conducted among all school-going children and their parents or guardians. These strategies can help reduce the prevalence and morbidity of schistosomiasis. Additionally, we recommend more research in areas similar to Kawama in Ndola district to describe the epidemiology of schistosomiasis.

### What is known about this topic

- *Schistosomiasis is the second most common neglected tropical disease in sub-Saharan Africa;*
- *Sub-Saharan Africa accounts for 13% of the global population yet disproportionally harbors 90% of the global schistosomiasis cases*.

### What this study adds

- *The identification of pupils in grades four, six, and seven as being more likely to be infected with S. haematobium provides an avenue for targeted community interventions in Kawama township, Ndola, Zambia;*
- *The magnitude of the public health of schistosomiasis among pupils aged 5 to 17 years in Kawama township, Ndola, Zambia was 133 cases per 1000 pupils;*
- *Water contact activities like swimming propagates schistosomiasis transmission among pupils aged 5 to 17 years in Kawama township, Ndola, Zambia*.

## References

[1] Bogitsh BJ, Carter CE, Oeltmann TN (2019). Blood Flukes.

[2] Verjee MA (2019). Schistosomiasis: Still a Cause of Significant Morbidity and Mortality. Res Rep Trop Med.

[3] Conteh L, Engels T, Molyneux DH (2010). Socioeconomic aspects of neglected tropical diseases. Lancet.

[4] Mupakeleni UN, Nyarko KM, Ananias F, Nsubuga P, Ndevaetela EE (2017). Factors associated with Schistosomiasis outbreak at Omindamba primary school, Omusati region, Namibia: a case-control study. Pan Afr Med J.

[5] Manz KM, Kroidl I, Clowes P, Gerhardt M, Nyembe W, Maganga L (2020). *Schistosoma haematobium* infection and environmental factors in Southwestern Tanzania: A cross-sectional, population-based study. PLoS Negl Trop Dis.

[6] Senghor B, Diallo A, Sylla SN, Doucouré S, Ndiath MO, Gaayeb L (2014). Prevalence and intensity of urinary schistosomiasis among school children in the district of Niakhar, region of Fatick, Senegal. Parasit Vectors.

[7] Munisi DZ, Buza J, Mpolya EA, Kinung´hi SM (2016). Intestinal Schistosomiasis among Primary School children in Two On-Shore Communities in Rorya District, Northwestern Tanzania: Prevalence, Intensity of Infection and Associated Risk Factors. J Parasitol Res.

[8] Karunamoorthi K, Almalki M, Ghailan KY (2018). Schistosomiasis: A neglected tropical disease of poverty: A call for intersectoral mitigation strategies for better health. J Heal Res Rev.

[9] Grimes JE, Croll D, Harrison WE, Utzinger J, Freeman MC, Templeton MR (2015). The roles of water, sanitation and hygiene in reducing schistosomiasis: A review. Parasit Vectors.

[10] Adeneye A, Sulyman M, Akande D, Mafe M (2021). Factors promoting schistosomiasis infection in endemic rural communities of Ifedore and Ile-Oluji/Oke Igbo local government areas in Ondo State, Nigeria. Glob J Infect Dis Clin Res.

[11] Inobaya MT, Olveda RM, Chau TN, Olveda DU, Ross AG (2014). Prevention and control of schistosomiasis: a current perspective. Res Rep Trop Med.

[12] Atalabi TE, Lawal U, Ipinlaye SJ (2016). Prevalence and intensity of genito-urinary schistosomiasis and associated risk factors among junior high school students in two local government areas around Zobe Dam in Katsina State, Nigeria. Parasit Vectors.

[13] Opara KN, Wilson EU, Yaro CA, Alkazmi L, Udoidung NI, Chikezie FM (2016). Prevalence, Risk Factors, and Coinfection of Urogenital Schistosomiasis and Soil-Transmitted Helminthiasis among Primary School Children in Biase, Southern Nigeria. Parasit Vectors.

[14] Chipeta MG, Ngwira B, Kazembe LN (2013). Analysis of *Schistosoma haematobium* Infection Prevalence and Intensity in Chikhwawa, Malawi: An Application of a Two Part Model. PLoS Negl Trop Dis.

[15] McManus DP, Dunne DW, Sacko M, Utzinger J, Vennervald BJ, Zhou XN (2018). Schistosomiasis. Nat Rev Dis Primers.

[16] Mbabazi PS, Andan O, Fitzgerald DW, Chitsulo L, Engels D, Downs JA (2011). Examining the Relationship between Urogenital Schistosomiasis and HIV Infection. PLoS Negl Trop Dis.

[17] Kalinda C, Chimbari MJ, Mukaratirwa S (2018). Schistosomiasis in Zambia: A systematic review of past and present experiences. Infect Dis Poverty.

[18] Ministry Of Health, Zambia (2019). Kawama clinic statistics.

[19] (2010). Central Statistical Office, Zambia City population: Kawama ward in Zambia.

[20] (2018). Schistosomiasis in School Going Children in Newly Established Transmission Site of Chifubu Area of Ndola Town, Zambia. Int J Curr Innov Adv Res Cit.

[21] Pourhoseingholi MA, Vahedi M, Rahimzadeh M (2013). Sample size calculation in medical studies, Gastroenterol Hepatol from Bed to Bench. Gastroenterol Hepatol Bed Bench.

[22] Chomba C, Mutale S (2014). Factors Characterising High Prevalence Rates of Urinary Schistosomiasis in Mufumbwe District, North Western Province of Zambia. Glob J Biol Agric Heal Sci.

[23] Mwanakasale V, Tente C, Chungu J, Xu J, Zhou X (2017). Inguinal Hernia: A probable complication of urinary schistosomiasis in school age male children in an area highly endemic for *Schistosoma haematobium* in Zambia. J Prev Med Care.

[24] Degarege A, Mekonnen Z, Levecke B, Legesse M, Negash Y, Vercruysse J (2015). Prevalence of *Schistosoma haematobium* Infection among School-Age Children in Afar Area, Northeastern Ethiopia. PLoS One.

[25] Abdulkareem BO, Habeeb KO, Kazeem A, Adam AO, Samuel UU (2018). Urogenital Schistosomiasis among Schoolchildren and the Associated Risk Factors in Selected Rural Communities of Kwara State, Nigeria. J Trop Med.

[26] Anto F, Asoala V, Adjuik M, Anyorigiya T, Oduro A, Akazili J (2013). Water Contact Activities and Prevalence of Schistosomiasis Infection among School-age Children in Communities along an Irrigation Scheme in Rural Northern Ghana. J Bacteriol Parasitol.

[27] Njunda AL, Ndzi EN, Assob JC, Kamga HL, Kwenti ET (2017). Prevalence and factors associated with urogenital schistosomiasis among primary school children in barrage, Magba sub-division of Cameroon. BMC Public Health.

[28] Sady H, Al-Mekhlafi HM, Atroosh WM, Al-Delaimy AK, Nasr NA, Dawaki S (2015). Knowledge, attitude, and practices towards schistosomiasis among rural population in Yemen. Parasit Vectors.

[29] Mohammed Hamad MN, Elfaki TM, Zarrug E, Musa Mohammed HO, Mohammed SH, Haj Ahmad RA (2020). Prevalence of schistosomiasis among school aged children in Altakamol area, Khartoum state, Sudan. J Microbiol.

[30] Mazigo HD, Nuwaha F, Kinung´Hi SM, Morona D, De Moira AP, Wilson S (2012). Epidemiology and control of human schistosomiasis in Tanzania. Parasit Vectors.

[31] Hajissa K, Muhajir AE, Eshag HA, Alfadel A, Nahied E, Dahab R, Ali SM, Mohammed M, Gaafar M, Mohamed Z (2018). Prevalence of schistosomiasis and associated risk factors among school children in Um-Asher Area, Khartoum, Sudan. BMC Res Notes.

[32] Gazzinelli A, Velasquez-Melendez G, Crawford SB, LoVerde PT, Correa-Oliveira R, Kloos H (2006). Socioeconomic Determinants of Schistosomiasis in a Poor Rural Area in Brazil. Acta Trop.

[33] Umoh NO, Nwamini CF, Inyang NJ, Umo AN, Usanga VU, Nworie A (2020). Prevalence of urinary schistosomiasis amongst primary school children in Ikwo and Ohaukwu Communities of Ebonyi State, Nigeria. Afr J Lab Med.

[34] Geleta S, Alemu A, Getie S, Mekonnen Z, Erko B (2015). Prevalence of urinary schistosomiasis and associated risk factors among Abobo Primary School children in Gambella Regional State, southwestern Ethiopia: A cross sectional study. Parasit Vectors.

[35] Angora EK, Boissier J, Menan H, Rey O, Tuo K, Touré AO (2019). Prevalence and Risk Factors for Schistosomiasis among Schoolchildren in two Settings of Côte d´Ivoire. Trop Med Infect Dis.

[36] Dawaki S, Al-Mekhlafi HM, Ithoi I, Ibrahim J, Abdulsalam A, Ahmed A (2016). Prevalence and risk factors of schistosomiasis among Hausa communities in Kano state, Nigeria. Rev Inst Med Trop Sao Paulo.

[37] Ndukwe YE, Obiezue RN, Aguzie IO, Anunobi JT, Okafor FC (2019). Mapping of urinary schistosomiasis in Anambra State, Nigeria. Ann Glob Health.

